# Simulating learning methodology (SLeM): an approach to machine learning automation

**DOI:** 10.1093/nsr/nwae277

**Published:** 2024-08-08

**Authors:** Zongben Xu, Jun Shu, Deyu Meng

**Affiliations:** School of Mathematics and Statistics, Xi’an Jiaotong University, China; Ministry of Education Key Lab of Intelligent Networks and Network Security, Xi’an Jiaotong University, China; Peng Cheng Laboratory, China; Pazhou Laboratory (Huangpu), China; School of Mathematics and Statistics, Xi’an Jiaotong University, China; Ministry of Education Key Lab of Intelligent Networks and Network Security, Xi’an Jiaotong University, China; Peng Cheng Laboratory, China; Pazhou Laboratory (Huangpu), China; School of Mathematics and Statistics, Xi’an Jiaotong University, China; Ministry of Education Key Lab of Intelligent Networks and Network Security, Xi’an Jiaotong University, China; Peng Cheng Laboratory, China; Pazhou Laboratory (Huangpu), China

## Abstract

This paper introduces a ‘simulating learning methodology’ (SLeM) approach for the learning methodology determination in general and for Auto^6^ ML in particular, and reports the SLeM framework, approaches, algorithms and applications.

## MACHINE LEARNING REQUIRES AUTOMATION

Machine learning (ML) is a fundamental technology of artificial intelligence (AI) that focuses on searching the possibly existing mapping $f:\mathcal {X}\rightarrow \mathcal {Y}$ to fit a given dataset $\mathcal {D}= \lbrace (x_i,y_i)\rbrace _{i=1}^N$, where each $(x,y)\in \mathcal {X}\times \mathcal {Y} \subset \mathbb {R}^d \times \mathbb {R}$. The traditional learning paradigm of ML research is to find a mapping $f^{*}$ from a predefined hypothesis space $\mathcal {F} = \lbrace f_{\theta }:\mathcal {X}\rightarrow \mathcal {Y}, \theta \in \Theta \rbrace$ by solving the following equation, based on a given optimality criterion (i.e. a loss function $\ell :\mathcal {Y} \times \mathcal {Y} \rightarrow \mathbb {R}^+$):


(1) \begin{eqnarray*}
f^{*} &=& \mathop {\arg \min }_{f_{\theta } \in \mathcal {F}} \mathbb {E}_{\mathcal {D}}[\ell (f_{\theta }(x),y)]\\
&&+\, \lambda R(f_{\theta }).
\end{eqnarray*}

Here $\mathbb {E}_{\mathcal {D}}$ denotes expectation with respect to $\mathcal {D}$, $R(\cdot )$ is a regularizer that controls the property of the solution and $\Theta$ is the set of parameters $\theta \in \mathbb {R}^p$.

Under such a learning paradigm, ML techniques like deep learning have revolutionized various fields of AI, e.g. computer vision, natural language processing and speech recognition, by effectively addressing complex problems that were once considered intractable. However, the effectiveness of ML always highly relies on some prerequisites of ML’s fundamental components before solving the aforementioned formulation. Some examples are as follows.

- *The **independence hypothesis** on the loss function.* The loss function $\ell$ is preset before implementation, certainly independent of the data distribution and application problems.
- *The **large capacity hypothesis** on the hypothesis space.* The hypothesis space $\mathcal {F}$ should be large enough in capacity to embody the optimal solution to be found. It is certainly preset independent of the application problems.
- *The **completeness hypothesis** on training data.* Samples $(x,y)$ in the training dataset should be well labeled, of low-level noise, class balanced and of sufficient number.
- *The **prior determination hypothesis** on the regularizer.* Regularizer *R* is fixed and preset by a prior of the hypothesis space $\mathcal {F}$, while only hyperparameter $\lambda$ is adjusted.
- *The **Euclidean space hypothesis** on the analysis tool.* The performance of ML can be analyzed in the Euclidean space, which means that the optimization algorithm (i.e. $\arg \min$) of solving parameters $\theta$ can always be naturally embedded in $\mathbb {R}^p$ with the Euclidean norm.

All of these prerequisites are standard settings in ML research. They can be seen as both the prompt for the rapid development of ML and the restraints on its progress. To improve the performance of existing AI technologies, it is necessary to break through these prior hypotheses of ML. However, it is easy to observe that we can optimally set these components if and only if the optimal solution to the problem is known in advance, falling into a ‘chicken or egg’ dilemma. Therefore, it is fundamental to establish some kind of best-fitting strategies to ML setting-up for ML applications. Recently, there have been a series of strategies towards breaking through these hypotheses of ML with a best-fitting theory, e.g. model-driven deep learning for large capacity/regularizer hypotheses, the noise modeling principle for the independence hypothesis, self-paced learning for the completeness hypothesis, Banach space geometry for the Euclidean hypothesis (see [1] and the references therein).

Though these strategies have demonstrated effectiveness and powerfulness, they still highly rely on manually presetting, not automatically designing purely from data. Specifically, at the data level, we still rely on human effort to collect, select and annotate data. Humans must determine which data should be used for training and testing purposes. At the model and algorithm level, we have to manually construct the fundamental structure of learning models (e.g. deep neural networks), predefine the basic forms of loss functions, determine the algorithm types and their hyperparameters of the optimization algorithms, etc. Moreover, at the task and environment level, current techniques are good at solving single tasks in a closed environment, while they are limited in handling complex and varying multi-tasks in a more realistic open and evolutionary environment. In a nutshell, the current learning paradigm that relies on extensive manual interventions on ML’s components struggles to handle complex data and diverse tasks in the real world, resulting in a degradation and unsatisfied learning capability of current ML techniques.

A natural approach to address the aforementioned challenges is to reduce manual interventions in the ML process via some learning strategies towards automation of ML. In other words, we hope to design ML’s fundamental components for enhancing adaptive learning capabilities of ML in an open and evolutionary environment with diverse tasks, thereby achieving the so-called machine learning automation (Auto$^6$ML)[1]. We can summarize Auto$^6$ML as the following six automation goals.

- *Data and sample level*: automatically generate data and select samples.
- *Model and algorithm level*: automatically construct models/losses and design algorithms.
- *Task and environment level*: automatically transfer between varying tasks and adapt to dynamic environments.

**Figure 1.**
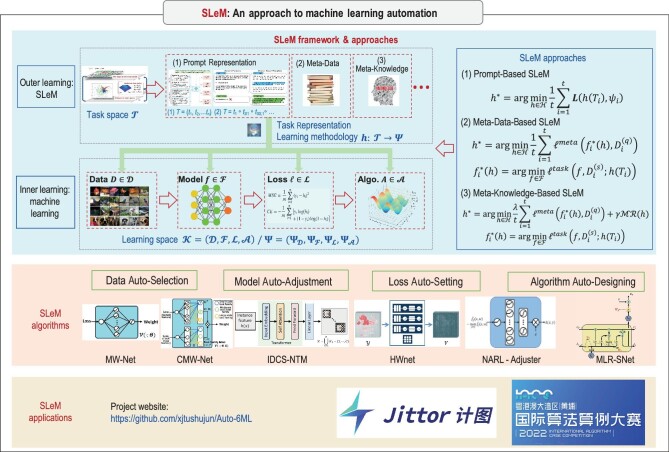
Illustration of the SLeM framework, theories, algorithms and applications for machine learning automation.

Achieving Auto$^6$ML could be understood as the automation regulation and design of ML’s fundamental components such as data, models, losses and algorithms, which intrinsically calls for a determination of ‘learning methodology’ mapping. In the following, we propose a ‘simulating learning methodology’ (SLeM) approach for the learning methodology determination in general and for Auto$^6$ML in particular. We report the SLeM framework, approaches, algorithms and applications in Fig. 1.

## SLeM FRAMEWORK AND APPROACHES

In this section, we propose a SLeM framework by formalizing the learning task, learning method and learning methodology, and then we present three possible computations to implement SLeM.

### Learning task

Machine learning summarizes the observable laws in the real world. From the view of mathematics and statistics, a learning task can thus be defined as a work that infers the underlying laws (i.e. probability density function) from observed data. In some sense, it is equivalent to a statistical inference task, and its specific forms include classification, regression, clustering, dimensionality reduction, etc. A learning task could be represented in many different ways; for example, (i) a task can be described by prompts via natural language instructions/demonstrations [2], i.e. $T=(t_1,\dots ,t_n)$, where $t_i$ is the task demonstration. The current popular large language model (LLM) solves the problem just via text prompt interaction with the model. Moreover, a task can be decomposed into a series of sub-tasks, i.e. $T = t_1 \circ t_{2|1} \circ t_{3|2,1} \circ \cdots$. Such a hierarchical prompt representation of a learning task can help solve a complicated reasoning task for LLM [3]. (ii) A task can be characterized by small-size high-quality data, called meta-data [4], denoted $D^{(q)}= \lbrace (x_i^{(q)}, y_i^{(q)})\rbrace _{i=1}^m$, which is popularly used in meta learning. (iii) A task can also be defined by a set of logic rules/knowledge, called meta-knowledge [5], which could also be utilized to quantify the task representation. More forms of task representation are still required, and the research on the precise mathematical formulation of a learning task is still ongoing.

### Learning method

We define a learning method as a specification of all four elements of ML in equation (1). More precisely, we define the learning space $\mathcal {K}=(\mathcal {D},\mathcal {F},\mathcal {L}, \mathcal {A})$, where $\mathcal {D},\mathcal {F},\mathcal {L}, \mathcal {A}$ denote the data (distribution functions), model (hypothesis), loss (loss functions) and algorithm spaces, respectively, and we define a learning method as an element in $\mathcal {K}$ when a learning task is given, with $\mathcal {D},\mathcal {F},\mathcal {L}, \mathcal {A}$ representing a proper data scheme, a learner’s architecture, a specific loss function and an optimization algorithm, respectively. The determination of the learning method could be considered as designing ML’s components for the task, which is potentially hopeful to alleviate the above ML prerequisites. To make the computation tractable, we suppose that $\mathcal {K}$ is separable, that is, each element in the learning space $\mathcal {K}$ can be expanded with a countably infinite number of base functions, and then $\mathcal {K}$ could be represented by the product of four infinite sequence spaces $\Psi = (\Psi _{\mathcal {D}}, \Psi _{\mathcal {F}}, \Psi _{\mathcal {L}}, \Psi _{\mathcal {A}})$. From this perspective, a learning method then corresponds to a hyperparameter assignment of $\mathcal {K}$. In other words, an effective hyperparameter configuration involved in the ML process can be interpreted as a proper ‘learning method’ imposed on a learning task [6]. In practice, we employ finite hyperparameter assignment sequences to approximate $\Psi$.

### Learning methodology

The learning methodology is a mapping from the task space $\mathcal {T}$ to the learning space $\mathcal {K}$ or $\Psi$, denoted $\mathcal {LM}:\mathcal {T}\rightarrow (\mathcal {K}$ or $\Psi )$. Thus, a learning methodology could be understood as a hyperparameter assignment rule of the learning method. Determination of the learning methodology is, however, an intrinsically infinite-dimensional ML problem.

### SLeM

SLeM aims to learn the learning methodology mapping $\mathcal {LM}$, or, in other words, learn the hyperparameter assignment rule of ML. To this end, we can employ an explicit hyperparameter setting mapping $h:\mathcal {T}\rightarrow \Psi$ conditioned on learning tasks that map from the learning task space $\mathcal {T}$ to the hyperparameter space $\Psi$, covering the whole learning process to simulate the ‘learning methodology’. Formally, we propose solving the following formulation to get the ‘learning methodology’ mapping *h* shared among various learning tasks:


(2) \begin{eqnarray*}
h = \mathop {\arg \min }_{h \in \mathcal {H}} \mathbb {E}_{(T,\psi ) \sim {\mathcal {S}} } \boldsymbol {L}(h(T),\psi ).\\
\end{eqnarray*}

Here $\boldsymbol {L}$ is a metric evaluating the learning method $\psi = (\psi _{\mathcal {D}}, \psi _{\mathcal {F}}, \psi _{\mathcal {L}}, \psi _{\mathcal {A}}) \in \Psi$ for learning task $T \in \mathcal {T}$, $\mathcal {S}$ is the joint probability distribution over $\mathcal {T}\times \Psi$ and $\mathcal {H}$ is the hypothesis space of *h*.

The obtained learning methodology mapping is promising to help ML model finely adapt to varying tasks from dynamic environments with fewer human interventions, and thereby achieving Auto$^6$ML. Note that the formulation in equation (2) is computationally intractable; a natural method to solve it is to collect observations $\lbrace (\mathcal {T}_i,\Psi _i)_{i=1}^t \rbrace$ from $\mathcal {S}$. We propose three typical realization approaches for SLeM according to different task representation forms, which are verified to be effective for achieving Auto$^6$ML in practice.

### Prompt-based SLeM

Suppose that we have access to observations $S = \lbrace (T_i, \psi _i)\rbrace _{i=1}^M$, denoted by task prompts and corresponding learning methods; then we can rewrite equation (2) as


(3) \begin{eqnarray*}
h = \mathop {\arg \min }_{h \in \mathcal {H}} \frac{1}{t} \sum _{i=1}^t \boldsymbol {L}(h(T_i),\psi _i).
\end{eqnarray*}

This approach is closely related to the recent LLM techniques [2]. When given a task prompt, the LLM directly predicts its solution, while SLeM firstly predicts its learning method, and then produces the solution based on the learning method. This understanding potentially reveals the insight of the task generalization ability of LLM techniques. However, such a ‘brute-force’ learning paradigm is cumbersome and labor intensive; how to develop lightweight-reduced implementations for this formulation is left for future study.

### Meta-data-based SLeM

Suppose that we have enough meta-data $D_i^{(q)}$ that can be used to properly evaluate learning methods adapting to learning task $T_i$; then we can rewrite equation (2) as


(4) \begin{eqnarray*}
&&\!\!\!\! h = \mathop {\arg \min }_{h \in \mathcal {H}} \frac{1}{t} \sum _{i=1}^t \ell ^{meta}\Big(f^{*}_{i}(h) ,D_i^{(q)}\Big)\\
&&\text{with}\\
&&\!\! f^{*}_{i}(h) = \mathop {\arg \min }_{f \in \mathcal {F}} \ell ^{task} \Big(f, D_i^{(s)}; h(T_i)\Big), \\
\end{eqnarray*}

where $\ell ^{meta}\ {\rm and}\ \ell ^{task}$ are meta and task losses, respectively, $\ell (f ,D) = (\frac{1}{|D|})\sum _{i=1}^{|D|} \ell (f(x_i), y_i)$ and $f^{*}_{i}(h)$ is the optimal learner for task $T_i$ given hyperparameter configurations predicted by $h(T_i)$. To better distinguish *f* and *h*, we usually call *h* a meta-learner. Here $D_i^{(s)}$ is the training set for task $T_i$, and we drop its explicit dependence on $h(T_i)$. Formulation (4) can be very easily integrated into the traditional ML framework to provide a fresh understanding and extension of the original ML framework. In the next section, we further show that such a meta-data-based SLeM formulation could greatly enhance adaptive learning capabilities of existing ML methods. We have provided a statistical learning guarantee for the task transfer generalization ability of the so-obtained learning methodology in [6], which makes Auto$^6$ML directly tractable and more solid.

### Meta-knowledge-based SLeM

Collecting meta-data may be costly and difficult in some applications. Instead, we also suggest utilizing meta-knowledge to evaluate the learning methodology [5]. Specifically, we propose the following meta-regularization (MR) approach for computing the learning methodology *h*:


(5) \begin{eqnarray*}
&& h = \mathop {\arg \min }_{h \in \mathcal {H}} \frac{\lambda }{t} \sum _{i=1}^t \ell ^{meta}\Big(f^{*}_{i}(h) ,D_i^{(q)}\Big) \\
&&\quad \ +\, \gamma \mathcal {MR}(h)\ \text{with} \\
&& f^{*}_{i}(h) = \mathop {\arg \min }_{f \in \mathcal {F}} \ell ^{task} \Big(f, D_i^{(s)}; h(T_i)\Big).\\
\end{eqnarray*}

Here $\mathcal {MR}(h)$ is a meta-regularizer that confines the meta-learner functions in terms of data augmentation consistency (DAC), regulated by meta-knowledge, and $\lambda , \gamma \ge 0$ are hyperparameters making a trade-off between meta-loss and the meta-regularizer. In [5], we theoretically showed that the DAC-MR approach can be treated as a proxy meta-objective used to evaluate the meta-learner without high-quality meta-data (i.e. $\lambda =0, \gamma > 0$). Besides, meta-loss combined with the DAC-MR approach is capable of achieving better meta-level generalization (i.e. $\lambda > 0, \gamma > 0$). We also empirically demonstrated that the DAC-MR approach could learn well-performing meta-learners from training tasks with noisy, sparse or even unavailable meta-data, well aligned with theoretical insights.

### Remark

The learning process of SLeM contains meta-training and meta-test stages, respectively. In the meta-training stage, we extract the learning methodology from given meta-training tasks. However, it often still needs human interventions to help get the learning methodology, like collecting meta-training tasks, designing the architecture of the learning methodology mapping, configuring hyperparameters of meta-training algorithms, etc. Yet we want to emphasize that, in the meta-test stage, our meta-learned learning methodology is fixed, which could be used to tune hyperparameters of ML in a plug-and-play manner. In this sense, it should be more rational to say that such a SLeM scheme alleviates the work load of tune additional hyperparameters of machine learning at the meta-test stage of SLeM, and thus potentially achieves Auto$^6$ML at the data and sample and model and algorithm levels. It is essential to note that SLeM still requires a human to specify what problem or task they want ML to solve, and to set input task information for the learning methodology mapping. When task information specified by users reflects the characteristic of varying tasks, the learning methodology could adaptively predict the machine learning method for varying tasks. In this sense, SLeM is potentially effective for addressing varying tasks from dynamic environments. In other words, SLeM could achieve Auto$^6$ML at the task and environment level with proper task information specified by a human.

## SLeM ALGORITHMS AND APPLICATIONS

Based on the proposed SLeM framework, we can readily develop a series of SLeM algorithms for Auto$^6$ML, as presented in the following. It is worth emphasizing that the realizations of Auto$^6$ML are mainly based on meta-data-based SLeM approaches in this paper.

### Data auto-selection

We explore the assignment of a weight $v_i \in [0,1]$ to each candidate datum $x_i$, which represents the possibility of $x_i$ being selected. Compared with conventional methods using pre-defined weighting schemes to assign values of the $v_i$, we adopt an MLP net called MW-Net [4] to learn an explicit weighting scheme. It has been substantiated that weighting functions automatically extracted from data comply with those proposed in the hand-designed studies for class imbalance or noisy labels. We further reform MW-Net by introducing a task feature as the supplementary input information, denoted CMW-Net [7], for addressing real-world heterogeneous data bias. CMW-Net is substantiated to be performable in different complicated data bias cases, and helps improve the performance of sample selection and label correction in a series of data bias issues, including datasets with class imbalance, different synthetic label noise forms and real-life complicated biased datasets. The meta-learned weighting scheme can especially be used in a plug-and-play manner, and can be directly deployed on unseen datasets, without needing to specifically tune extra hyperparameters of the CMW-Net algorithm.

### Model auto-adjustment

The existing backbone networks have limited ability to adapt to different distribution shifts. They always use a noise transition matrix to adjust the prediction of the deep classifier for addressing the influence of noisy labels. Compared with previous methods specifically designed based on knowledge of the transition matrix, we use a transformer network, called IDCS-NTM [8], to automatically predict the noise transition for adjusting the prediction of the deep classifier adapting to various noisy labels. Meanwhile, the meta-learned noise transition network can help adjust the prediction of the deep classifier on unseen real noisy datasets, and achieves better performance compared with manually designed noise transition.

### Loss auto-setting

For a regression task, the form of the loss function corresponds to the distribution of the underlying noise. How to set the loss function could be formulated as a weighted loss optimization problem. Conventional methods attempt to solve weighted loss by assigning the unknown distribution subjectively or fixing the weight vector empirically, which makes them hard to address complex scenarios adaptively and effectively. We use a hyper-weight network (HWnet) [9] to predict the weight vector. HWnet could automatically adjust weights for different learning tasks, so as to auto-set the loss function in compliance with the tasks at hand. The meta-learned HWnet can be explicitly plugged into other unseen tasks to finely adapt to various complex noise scenarios, and helps improve their performance. For the classification task, we also explore a loss adjuster [10] to automatically set robust loss functions of every instance for various noisy label tasks. The meta-learned loss adjuster could also transfer to unseen real-life noisy datasets, and achieves better performance compared with hand-designed robust loss functions with carefully tuned hyperparameters.

### Algorithm auto-designing

The stochastic gradient descent algorithm requires manually presetting a learning rate (LR) schedule (i.e. $\lbrace \alpha _t\rbrace _{t=1}^T$ with *T* the total iteration steps) for the task at hand. We use a long short-term memory-based net, called MLR-SNet [11], to adaptively set the LR schedule. MLR-SNet could automatically learn a proper LR schedule to comply with the training dynamics of different deep neural network (DNN) training problems, which are more flexible than hand-designed policies for specific learning tasks. The meta-learned LR schedule is plug and play, and could be readily transferred to unseen heterogeneous tasks. MLR-SNet is substantiated to be transferable among DNN training tasks of different training epochs, datasets and network architectures and the large-scale ImageNet, and achieves comparable performance with the corresponding best hand-designed LR schedules in the test data.

### SLeM applications

We have released the aforementioned SLeM algorithms on an open-source platform at https://github.com/xjtushujun/Auto-6ML based on Jittor, aiming to provide a toolkit box for users to handle real-life Auto$^6$ML problems. Recently, our CMW-Net algorithm [12] was the champion of the 2022 International Algorithm Case Competition, which achieves a competitive sample selection and label correction performance on real-life heterogeneous and diverse label noise tasks, showing its potential usefulness for more practical datasets and tasks. It is possible to utilize SLeM algorithms to real application problems possessing features of varying multiple tasks from dynamic environments. For example, the visual unmanned navigation problem calls for reliable feature extraction and matching techniques that generalize to different geophysical scenarios and multimodal data; the smart education problem calls for effective visual recognition, detection and analysis techniques that generalize to diverse teaching scenarios and analysis tasks; and so on.

## RELATED FIELDS

AutoML [13,14] encompasses a wide range of methods aiming to automate traditionally manual aspects of the machine learning process, such as data preparation, algorithm selection, hyperparameter tuning and architecture search, while it has limited researches on automatical transfer between varying tasks, which is emphasized by aforementioned Auto$^6$ML. Existing AutoML methods are mostly heuristic, making it difficult to develop theoretical evidence. In comparison, our SLeM framework establishes a unified mathematical formulation for Auto$^6$ML, and provides theoretical insight into the task transfer generalization ability of SLeM [6].

Algorithm selection [15] learns a mapping from the problem space to the algorithm space by searching for the optimal algorithm from a pool of finite algorithms for the tasks at hand. It is usually inflexible to addressing varying tasks. The SLeM adopts bi-level optimization tools to extract learning methodology mapping for predicting the proper learning method of different tasks with a sound theoretical guarantee, which could more flexibly and adaptively fit query tasks.

## CHALLENGES

Existing SLeM algorithms only realize automation for each component of ML, which is far from the goal of Auto$^6$ML. In particular, the learning process of SLeM still requires extensive human interventions and selections. Achieving SLeM algorithms with stronger automation capabilities and more complex automation problems/scenarios is still an important problem in future research. Moreover, developing the lightweight prompt-based SLeM approach is worth deeper and more comprehensive exploration for the reduction of the LLM. Besides, we try to construct a novel learning theory on infinite-dimensional functional space to finely reveal the insights of SLeM, and develop task-generalized transfer learning theory to provide a theoretical foundation for handling varying tasks and dynamic environments in real-world applications. Building connections between SLeM and other techniques on exploring task-transferable generalization, like meta-learning, in-context learning and large foundation models, is also valuable for future research.
